# Severe Undernutrition Predicts Adverse Outcomes in Transplant Norovirus Infection

**DOI:** 10.1016/j.ekir.2026.106679

**Published:** 2026-06-24

**Authors:** Ivan Scriabine, Julien Zuber, Claire Deback, Hélène François, Marie Matignon, Renaud Snanoudj, Dany Anglicheau, Mohamad Zaidan, Manon Dekeyser

**Affiliations:** 1 Department of Nephrology-Dialysis-Transplantation, Bicêtre Hospital, Assistance Publique des Hôpitaux de Paris, Le Kremlin-Bicêtre, France; 2 Département des Maladies du Rein et du Métabolisme, Transplantation et Immunologie Clinique, Hôpital Necker, Assistance Publique des Hôpitaux de Paris, Université Paris Cité, Paris, France; 3 Department of Inflammation, Microbiome et Immunosurveillance, Institut National de la santé et de la Recherche Médicale (INSERM), Université Paris-Saclay, Orsay, France; 4 Laboratoire de Virologie, Assistance Publique-Hôpitaux de Paris, Hôpitaux Universitaires Paris-Saclay, Hôpital Ambroise Paré, Boulogne Billancourt, France; 5 Division of Renal Transplantation, Department of Nephrology, Hôpital Pitié-Salpêtrière, Assistance Publique des Hôpitaux de Paris, Institut National de la santé et de la Recherche Médicale (INSERM) 1155, Sorbonne University, Paris, France; 6 Department of Nephrology, Henri Mondor Hospital, Assistance Publique des Hôpitaux de Paris, Créteil, France; 7 Immunology and Histocompatibility Laboratory, Saint-Louis Hospital, Assistance Publique des Hôpitaux de Paris, Paris, France; 8 Paris-Saclay University, Le Kremlin-Bicêtre, France; 9 Centre de Recherche en Immunologie des Infections Virales et des Maladies Auto-Immunes, Institut National de la Santé et de la Recherche Médicale U1184 Le Kremlin-Bicêtre, France; 10 Department of Nephrology, Centre Hospitalier Universitaire d’Orléans, Orléans University, Orléans, France; 11 Laboratoire Interdisciplinaire pour l’Innovation et la Recherche en Santé d’Orléans (LI2RSO) – Equipe 1: Thérapies Innovantes et Nanomédecine, Institut National de la santé et de la Recherche Médicale (INSERM) ART-ARNm US55, Orléans, France

**Keywords:** kidney transplant recipients, norovirus, undernutrition

## Abstract

**Introduction:**

Norovirus infection is a frequent cause of diarrhea after kidney transplantation, with heterogeneous clinical presentations. Without specific antiviral treatment, management mainly relies on minimization of immunosuppression. However, prognostic factors for mortality and graft loss remain poorly defined.

**Methods:**

We performed a multicenter retrospective study of kidney transplant recipients (KTRs) with norovirus infection between January 2017 and December 2022. A multicentric matched control group of 96 KTRs with nonnorovirus diarrhea was included for comparative nutritional analysis. Variables were collected at baseline, diagnosis, and follow-up, and included recipient or donor characteristics, infection features, immunosuppressive adjustment, nutritional status, and outcomes. Factors associated with mortality and graft loss were analyzed using univariate and multivariate models.

**Results:**

A total of 159 KTRs with norovirus infection were followed-up for a median of 2.7 years. At diagnosis, the norovirus group showed a significantly greater decrease in body mass index (BMI) than the controls (−5.5% vs. −1.5%, *P* < 0.0001), along with a higher prevalence of severe undernutrition (29.6% vs. 10.4%, *P* = 0.0010). Mortality (7.3 events/100 person-years) and graft loss (4.4 events/100 person-years) increased stepwise with the severity of undernutrition. Severe undernutrition at diagnosis was independently associated with both mortality (hazard ratio [HR] = 3.87 [1.34–11.21], *P* = 0.0126) and graft loss (HR = 2.96 [1.02–8.60], *P* = 0.0468), along with age, diabetes, and baseline creatininemia. Despite immunosuppressive minimization, no significant improvement in survival outcomes was observed.

**Conclusion:**

Severe undernutrition at diagnosis is a major independent predictor of adverse outcomes in KTRs with norovirus infection. Therefore, we recommend close monitoring of nutritional parameters and active nutrition strategies to prevent severe undernutrition in this high-risk population.

## Graphical abstract

**Figure:**
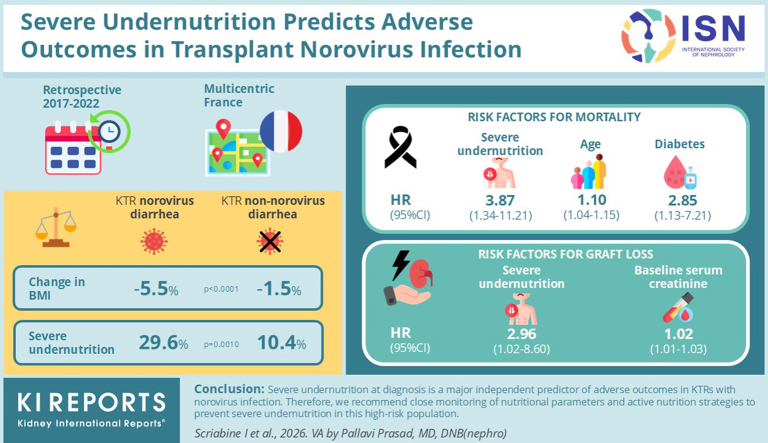

Kidney transplantation has become the gold-standard treatment for end-stage kidney disease, improving both quality of life and patient survival compared with dialysis. Nevertheless, long-term immunosuppression increases susceptibility to infections, including gastrointestinal infections.^1^ Among KTRs, chronic diarrhea is frequent, affecting ≤50%, and is often multifactorial.^2^

Over the past decade, noroviruses, which are nonenveloped, single-stranded positive-sense RNA viruses from the Caliciviridae family, have emerged as a major cause of chronic diarrhea in immunocompromised individuals, particularly after solid organ transplantation.^3^ Molecular studies have confirmed the broad genomic diversity of noroviruses, with new variants regularly replacing previously predominant strains.^4^ Ten genogroups have been described, of which genogroups I, II, and IV are pathogenic in humans.^5^ These viruses are highly contagious via the fecal-oral route and are the most common cause of acute gastroenteritis worldwide.^6^ Diagnosis relies on stool polymerase chain reaction (PCR) testing.^7^

In healthy individuals, norovirus infection is typically self-limiting, causing brief episodes of diarrhea, vomiting, and abdominal pain. The infection usually resolves within a few days and may require only transient symptomatic treatment.

In contrast, norovirus infection after transplantation can be more severe. KTRs may develop chronic or relapsing diarrhea, prolonged viral shedding (lasting months to years), and significant clinical consequences, including dehydration, undernutrition, and weight loss. Acute kidney injury is common (23%–81%), potentially compromising kidney graft survival.^8^, ^9^, ^10^, ^11^, ^12^

In the absence of a specific antiviral treatment,^13^^,^^14^ the current strategy relies on supportive measures, including rehydration and symptomatic management, as well as reduction of the immunosuppressive treatment.^2^^,^^3^^,^^15^ However, immunosuppression minimization may lead to the development of *de novo* donor-specific antibodies and an increased risk of allograft rejection.^16^ Alternative therapies, such as nitazoxanide, polyclonal immunoglobulins, and fecal microbiota transplantation, have been proposed without robust evidence of efficacy.^17^, ^18^, ^19^, ^20^ To date, available guidelines for managing norovirus infection in immunocompromised hosts primarily recommend these supportive measures in the absence of specific validated antiviral therapies. Nevertheless, these recommendations remain mostly nonspecific or based on low-level evidence.^15^

Prognostic factors for mortality and allograft survival remain poorly defined.^11^^,^^12^^,^^16^ Although prior studies have addressed metabolic complications and bone disease,^21^ the prognostic impact of undernutrition on the course of norovirus infection has not been thoroughly investigated. In this multicentric retrospective study, we aimed to determine the predictive factors for patient and kidney allograft survival, with a specific focus on nutritional status and immunosuppressive treatment minimization.

## Methods

### Study Design

We conducted a retrospective multicentric study, including KTRs with norovirus infection between January 2017 and December 2022 in 4 French transplant centers (Assistance Publique-Hôpitaux de Paris: Bicêtre, Henri Mondor, Necker, and Tenon hospitals). The study complied with French ethical regulations and was classified as minimal-risk research. A declaration of conformity was submitted to the French data protection authority (CNIL: 2233629), and patient nonopposition was required for data use. In addition, we established a control group of KTRs presenting with diarrhea unrelated to norovirus infection. This group included all eligible KTRs with diarrhea and a negative norovirus PCR result, retrospectively identified within 2 transplant centers (Bicêtre Hospital and Henri Mondor Hospital), thereby adopting a multicentric design consistent with the norovirus cohort and improving comparability between groups. Although the group sizes were imbalanced, comparisons were performed using statistical tests appropriate for unequal sample sizes and nonnormally distributed data (i.e., Chi-square and Mann–Whitney U tests).

### Norovirus Infection Diagnosis

Diagnosis was based on compatible clinical features and stool detection of norovirus using a qualitative multiplex PCR (BIOFIRE FILMARRAY Gastrointestinal Panel).^22^ Clinical features included diarrhea (≥ 3 watery stools/d), duration of diarrhea (categorized as acute diarrhea if < 4 weeks, and chronic diarrhea if ≥ 4 weeks), recurrent vomiting, abdominal pain, asthenia, anorexia, and weight loss. Patients were routinely tested for bacterial (e.g., *Campylobacter spp., Yersinia spp., Shigella spp., Salmonella spp.*) and parasitic pathogens, as well as for *toxigenic Clostridium difficile* (toxin-B gene RT-PCR) in stool samples. In addition, cytomegalovirus (CMV) reactivation, assessed by blood CMV PCR performed on plasma samples, was defined as a viral load > 2 log copies/ml, in line with published definitions in KTRs.

### Definition of Nutritional Status

Nutritional status was classified according to the 2021 guidelines of the French Authority for Health (Haute Autorité de Santé). For patients aged <70 years, normal nutritional status was defined as BMI >18.5 kg/m^2^, stable BMI or <10% BMI loss from baseline, and serum albumin >35 g/l; moderate undernutrition was defined by BMI of 17 to 18.5 kg/m^2^, or >10% BMI loss, or albumin of 30 to 35 g/l; and severe undernutrition was defined by BMI <17 kg/m^2^, or >15% BMI loss, or albumin <30 g/l. For patients aged ≥70 years, thresholds were higher: normal status required BMI >22 kg/m^2^, stable BMI or <10% BMI loss from baseline, and albumin >30 g/l; moderate undernutrition was defined by BMI of 20 to 22, or >10% BMI loss, or albumin >30 g/l; and severe undernutrition by BMI <20, or >15% BMI loss, or albumin <30 g/l (Supplementary Figure S1).

### Data Collection

Clinical data and outcomes were retrospectively extracted from electronic medical records. All biological data affected by patients’ hydration status were collected after correction of dehydration and restoration of euvolemia. The following variables were collected:

(i) Baseline characteristics (within the 3 months preceding diagnosis), defined as data collected at the most recent clinical assessment within the 3 months preceding diagnosis, included demographic features (age, weight, height, BMI, presence of diabetes mellitus, and primary kidney disease) and transplant-related characteristics (transplant rank; immunosuppressive therapy, including induction and maintenance regimens; history of rejection episodes; and baseline serum creatinine level [μmol/l]).
(ii) At norovirus infection diagnosis, included time since kidney transplantation, diarrhea duration (acute or chronic), weight loss, weight or BMI changes, nutritional status, immunosuppressive regimen, serum creatinine and albumin levels, occurrence and stage of acute kidney injury according to the 2020 Kidney Disease: Improving Global Outcomes guidelines,^23^ and microbiological differential diagnostics.
(iii) Follow-up data, collected at 3, 6, and 12 months after diagnosis included persistence of gastrointestinal symptoms, changes in weight, BMI, and serum creatinine levels (μmol/l), and therapeutic interventions: reduction of immunosuppression, use of adjuvant therapies (nitazoxanide, polyclonal Igs, ribavirin, or fecal microbiota transplantation), and symptomatic treatments (antidiarrheal agents, nutritional support).
(iv) Major clinical outcomes: biopsy-proven rejection (according to the Banff criteria), return to dialysis, and death were recorded.

### Statistical Analysis

Categorical variables were expressed as counts and percentages and compared using chi-square or Fisher exact test, as appropriate. Continuous variables, including age, BMI, and serum creatinine, were assessed for normality by visual inspection (histograms) and the Shapiro–Wilk test. Because most were not normally distributed, they were presented as medians with interquartile ranges (IQRs) and compared using Mann-Whitney–Wilcoxon or Kruskal-Wallis tests. Survival analysis was performed using Kaplan-Meier curves; for comparisons across nutritional status groups, we used the log-rank test for trend, reflecting the ordered severity (no, moderate, severe undernutrition). Death and return to dialysis were treated as competing events and analyzed using cumulative incidence functions. In addition, incidence rates (events per person-year) and incidence rate ratios with 95% confidence intervals (CIs) were calculated overall and by nutritional status using univariable Poisson regression models, after verification of Poisson model assumptions. Cox proportional hazards models were applied for univariable and multivariable analyses. Variables were included in the multivariable models if they had a *P*-value < 0.10 in univariable analysis. To minimize overfitting, we followed the rule of 10 events per variable and limited the number of covariates accordingly (≤3 for mortality, ≤2 for graft loss). The proportional hazards assumption was verified using Schoenfeld residuals. Collinearity between variables was assessed using variance inflation factors. A sensitivity analysis including transplant center as a random effect was performed to assess potential center-level variability. All tests were 2-sided, and *P*-values < 0.05 were considered statistically significant. Analyses used GraphPadPrism-8.4.3 and R++4.3.1 and all tests were 2-sided, and *P*-values < 0.05 were considered statistically significant.

## Results

### Study Population

A total of 159 KTRs with norovirus infection were included: 91 patients (57.2%) from Necker, 30 patients (18.9%) from Bicêtre, 25 patients (15.7%) from Tenon, and 13 patients (8.2%) from Mondor, without significant center-level effect. The median follow-up duration after norovirus infection was 2.7 (IQR: 1.3–4.0) years.

Baseline demographic and transplant characteristics, collected within 3 months before diagnosis, are presented in Table 1. Patients had a median age of 59 (IQR: 49–68) years, 57.2% were male, and 37.1% had diabetes. Median baseline BMI was 24.0 (IQR: 20.7–27.7) kg/m^2^, median estimated glomerular filtration rate (based on the Chronic Kidney Disease–Epidemiology Collaboration 2021) was 46.8 (IQR: 34.1–61.0) ml/min per 1.73 m^2^, and median serum creatinine level was 130 (IQR: 110–170) μmol/l. Induction therapy included antithymocyte globulins (45.3%) or basiliximab (53.5%). Maintenance immunosuppression consisted of triple therapy in 88.7% of patients, including corticosteroids (*n* = 150, 94.3%); calcineurin inhibitors (tacrolimus or ciclosporine) (*n* = 118, 74.2%) or belatacept (*n* = 36, 22.6%); and antimetabolites (mycophenolate mofetil, mycophenolic acid, or azathioprine) (*n* = 143, 89.9%) or mammalian target of rapamycin inhibitors (*n* = 13, 8.2%). The most frequent triple therapy combination was calcineurin inhibitors, antimetabolites, and corticosteroids in 95 patients (59.7%). Eighteen patients (11.3%) were on dual therapy. Details of immunosuppressive regimens are provided in Table 1.

**Table 1:** Initial and follow-up characteristics of kidney allograft recipients with norovirus infection

Baseline characteristics (within the 3 mos preceding diagnosis)	*N*	
Age (yrs)	159	59 (49–68)
Men	159	91 (57.2%)
Diabetes	159	59 (37.1%)
Baseline BMI (kg/m^2^)	149	24.0 (20.7–27.7)
Baseline eGFR (ml/min per 1.73 m^2^)	159	46.8 (34.1–61.0)
Baseline creatininemia (μmol/l)	159	130 (110–170)
Characteristics of kidney transplantation		
Induction immunosuppression		
Antithymocyte globulins	159	72 (45.3%)
Basiliximab	159	85 (53.5%)
Maintenance immunosuppression		
MMF/MPA or azathioprine	159	143 (89.9%)
CNI (tacrolimus or cyclosporine)	159	118 (74.2%)
Everolimus	159	13 (8.2%)
Belatacept	159	36 (22.6%)
Corticosteroids	159	150 (94.3%)
MMF/MPA or azathioprine - CNI - corticosteroids	159	95 (59.7%)
MMF/MPA or azathioprine - belatacept - corticosteroids	159	33 (20.8%)
MMF/MPA or azathioprine - everolimus - corticosteroids	159	5 (3.1%)
CNI - everolimus - corticosteroids	159	6 (3.8%)
Belatacept - everolimus - corticosteroids	159	2 (1.3%)
MMF/MPA or azathioprine – CNI	159	9 (5.7%)
CNI - corticosteroids	159	7 (4.4%)
Belatacept - corticosteroids	159	1 (0.6%)
Azathioprine - corticosteroids	159	1 (0.6%)
Rejection episodes	157	50 (31.8%)
Norovirus infection characteristics at diagnosis		
Delay between transplantation and diagnosis (yrs)	159	4.9 (1.77–9.8)
Early - Late infection^a^	159	30 (18.9%) – 129 (81.1%)
Clinical features		
Acute - Chronic diarrhea	150	92 (61.3%) – 58 (38.7%)
Abdominal pain	147	35 (23.8%)
Nausea/vomiting	147	42 (28.6%)
Symptoms duration (mos)	44	3 (1–5)
Diagnosis BMI (kg/m^2^)	126	22.1 (19.0–26.3)
Weight loss	134	101 (75.4%)
Decrease in BMI	126	5.5% (0.0%–10.6%)
Acute kidney injury	156	96 (61.5%)
KDIGO stage 1	156	73 (46.8%)
KDIGO stage 2	156	16 (10.3%)
KDIGO stage 3	156	7 (4.5%)
Nutritional status at diagnosis		
Normal	159	84 (52.8%)
Moderate undernutrition	159	28 (17.6%)
Severe undernutrition	159	47 (29.6%)
Associated CMV reactivation and gastrointestinal coinfections		
Extended microbiological assessment	159	122 (76.7%)
Positive extended microbiological assessment		34 (27.9%)
CMV DNAemia > 2 log p/ml		19 (55.9%)
CMV viral load		3.2 (2.9-3.8)
Toxinogenic *Clostridium difficile*		5 (14.7%)
Positive coproculture		7 (20.6%)
Positive parasitological examination of stool		3 (8.8%)
Therapeutic management of norovirus infection		
Immunosuppressive treatment		
No modification	159	39 (24.5%)
Immunosuppressant minimization	159	120 (75.5%)
Withdrawal of one molecule		28 (23.3%)
Replacement of one molecule by another		40 (33.3%)
Dose-reduction of one molecule		52 (43.3%)
Return to baseline immunosuppression at M3 following diagnosis	120	29 (24.2%)
Other therapeutics^b^	159	16 (10.1%)
Symptomatic therapeutics		
Antidiarrheals	159	86 (54.1%)
Nutritional supplements	159	12 (7.6%)
Long term follow-up and outcomes		
Follow-up duration after diagnosis (yrs)	159	2.7 (1.3–4.0)
Persistence of gastrointestinal symptoms > M3 following diagnosis	159	21 (13.2%)
BMI at M12 following diagnosis or last follow-up (kg/m^2^)	123	24.3 (21.1–27.4)
Patient survival		
Death from all causes	159	35 (22.0%)
Time to death after infection (yrs)	159	1.6 (0.8–2.5)
Kidney transplant function		
Returning to hemodialysis	159	21 (13.2%)
Time to resumption of hemodialysis after infection (yrs)	159	1.0 (0.5–1.7)
Return to baseline serum creatinine	151	107 (70.9%)
No return to baseline serum creatinine	151	44 (29.1%)
Relative increase of serum creatinine (μmol/l)	151	20.5 (−0.8 to 48.8)
Rejection	159	15 (9.4%)
Before M3 following diagnosis		7 (46.7%)
After M3 following diagnosis		8 (53.3%)

BMI, body mass index; CMV, cytomegalovirus; CNI, calcineurin inhibitor; eGFR, estimated glomerular filtration rate; IQR, interquartile range; KDIGO, Kidney Disease: Improving Global Outcomes; M3, month 3; M12. Month 12; MMF, mycophenolate mofetil; MPA, mycophenolic acid.

Values are given as number (percentages) or median (IQR).

a Before or after M12 of transplantation.

b Nitazoxanide, polyclonal i.v. Igs, ribavirin, fecal microbiota transplantation.

### Norovirus Infection Diagnosis

Norovirus infection was diagnosed a median of 4.9 (IQR: 1.8–9.8) years after transplantation, with 18.9% of cases occurring within the first posttransplant year. At diagnosis, 92 patients (61.3%) had acute diarrhea, whereas 58 (38.7%) had chronic diarrhea (≥ 4 weeks). In 77 KTRs, gastrointestinal symptoms, including nausea ro vomiting and abdominal pain, had been present for a median of 3 (IQR: 1–5) months before diagnosis and were associated with weight loss in 75.4% of these patients.

Extended microbiological assessment was performed in 122 patients (76.7%), with 34 (27.9%) testing positive. Identified gastrointestinal pathogens included toxigenic *C difficile* (*n* = 5; 14.7%), bacterial (*n* = 7; 20.6%) and parasitic infections (*n* = 3; 8.8%). In addition, CMV reactivation (defined as blood CMV PCR from plasma samples > 2 log copies/ml) was identified in 19 patients (11.9%), with a median viral load of 3.2 (IQR: 2.9–3.8) log copies/ml (Table 1).

### Nutritional Status Assessment at Norovirus Infection Diagnosis

**Figure 1:**
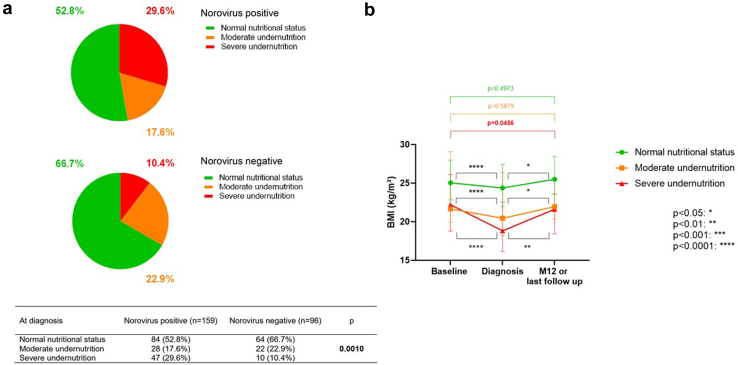
Nutritional status at diagnosis and its prognostic significance in kidney transplant recipients with norovirus infection. (a) Prevalence of nutritional status categories (normal, moderate, severe undernutrition) at the time of diagnosis in kidney transplant recipients with norovirus infection (*n* = 159) compared with a matched control group of kidney transplant recipients with diarrhea unrelated to norovirus (*n* = 96). At diagnosis, severe undernutrition was more frequent in the norovirus group (29.6%) than in controls (10.4%) (*P* = 0.0010). Comparisons were performed using Mann–Whitney tests for intergroup comparisons. (b) Evolution of BMI over time in kidney transplant recipients with norovirus infection (baseline, diagnosis, 3, 6, and 12 months) according to nutritional status at diagnosis. Patients with severe undernutrition exhibited the greatest BMI loss at diagnosis and the most limited nutritional recovery at follow-up, with a persistent deficit compared with baseline BMI (−4%, *P* = 0.0456). Values are presented as medians with interquartile ranges; comparisons used paired Wilcoxon test, as appropriate. BMI, body mass index; M12, month 12.

At diagnosis, median BMI was 22.1 (IQR: 19.0–26.3) kg/m^2^, representing a 5.5% (IQR: 0.0%–10.6%) decrease from baseline (*P* < 0.0001). Based on predefined criteria, 52.8% (*n* = 84) had normal nutritional status, 17.6% (*n* = 28) had moderate undernutrition, and 29.6% (*n* = 47) had severe undernutrition (Figure 1a). Comparison between patients with normal, moderate, or severe undernutrition at diagnosis revealed that baseline BMI was significantly different with respectively 25.04 (IQR: 22.8–28.0) kg/m^2^, 21.7 (IQR: 19.9–29.1) kg/m^2^, and 22.1 (IQR: 18.8–26.1) kg/m^2^, (*P* = 0.0038). Importantly, this difference was mainly driven by higher baseline BMI in patients with normal nutritional status, whereas no significant difference was observed between KTRs with moderate and severe undernutrition.

Patients with severe undernutrition had significantly lower BMI at norovirus diagnosis (18.8 [IQR: 16.2–22.6] vs. 20.5 [IQR: 18.2–26.4] and 24.4 [IQR 21.9–27.5] kg/m^2^, respectively; *P* < 0.0001,) and greater weight loss (10.6% [IQR: 4.4%–17.7%] vs. 5.3% [IQR: 0.0%–11.5%] and 3.8% [IQR: 0.0%–6.4%]; *P* < 0.0001, respectively), despite similar proportion of chronic diarrhea at diagnosis across groups (43.5% vs. 35.7% and 36.8%; *P* = 0.7210) (Figure 1b).

To assess whether nutritional impairment was specifically associated with norovirus infection, we compared the norovirus cohort to a control group of 96 KTRs with diarrhea unrelated to norovirus (including 65.3% of noninfectious diarrhea and 34.7% infectious diarrhea; Supplementary Table S1). The 2 groups were comparable for age, sex, diabetes, and baseline BMI (24.0 vs. 24.8 kg/m^2^, *P* = 0.1233) and survivals (Table 2). However, the norovirus group had a significantly lower BMI at diagnosis (22.1 [IQR: 19.0–26.3] vs. 24.6 [IQR 21.3–28.5] kg/m^2^, *P* = 0.0018), greater relative BMI decrease (−5.5% vs. −1.5%, *P* < 0.0001) (Table 2 and Supplementary Figure S2), and a higher rate of severe undernutrition (29.6% vs. 10.4%, *P* = 0.0010) (Figure 1a). These findings support a more pronounced nutritional impact of norovirus infection than other causes of diarrhea in KTRs. Nutritional status at diagnosis of diarrhea in nonnorovirus group was not significantly associated with impaired patient or graft survival (Supplementary Table S2).

**Table 2:** Comparative characteristics of kidney allograft recipients with norovirus and nonnorovirus-related diarrhea

Demographic features	Controls	Norovirus	*P*
Number of patients	96	159	
Age (yrs)	60 (50–65.8)	59 (49–68)	0.6475
Men	52 (54.2%)	91 (57.2%)	0.6964
Diabetes	39 (40.6%)	59 (37.1%)	0.5970
Baseline BMI (kg/m^2^)	24.8 (22.0–28.3)	24.0 (20.7–27.7)	0.1233
Characteristics of kidney transplantation			
Maintenance immunosuppression			
MMF/MPA or azathioprine - CNI - corticosteroids	65 (67.7%)	95 (59.8%)	0.2299
MMF/MPA or azathioprine - belatacept - corticosteroids	3 (3.1%)	33 (20.8%)	< 0.0001
MMF/MPA or azathioprine - everolimus - corticosteroids	2 (2.1%)	5 (3.1%)	0.7139
CNI - everolimus - corticosteroids	15 (15.6%)	6 (3.8%)	0.0012
Belatacept - everolimus - corticosteroids	0 (0%)	2 (1.3%)	0.5287
MMF/MPA or azathioprine – CNI	4 (4.2%)	9 (5.7%)	0.7717
CNI - corticosteroids	6 (6.3%)	7 (4.4%)	0.5635
Belatacept - corticosteroids	1 (1.0%)	1 (0.6%)	0.7222
Azathioprine - corticosteroids	0 (0%)	1 (0.6%)	> 0.9999
Rejection episodes	25 (26.0%)	50 (31.9%)	0.3949
Diarrhea characteristics at diagnosis			
Delay between transplantation and diagnosis (yrs)	3.5 (0.8–7.0)	4.9 (1.7–9.8)	0.0164
Clinical features			
Acute - chronic diarrhea	72 (75.0%) - 24 (25.0%)	92 (61.3%) - 58 (38.7%)	0.0372
Symptoms duration (mos)	2.0 (1.0–4.8)	3 (1–5)	0.5220
Abdominal pain	27 (28.4%)	35 (23.8%)	0.4528
Nausea/vomiting	24 (25.0%)	40 (27.2%)	0.7666
Diagnosis BMI (kg/m^2^)	24.6 (21.3–28.5)	22.1 (19.0–26.3)	0.0018
Decrease in BMI	1.5% (0.0%–5.5%)	5.5% (0.0%–10.6%)	< 0.0001
Nutritional status at diagnosis			
Normal	64 (66.7%)	84 (52.8%)	0.0010
Moderate undernutrition	22 (22.9%)	28 (17.6%)	
Severe undernutrition	10 (10.4%)	47 (29.6%)	
Follow-up			
Follow-up duration after diagnosis (yrs)	3.1 (1.8–4.3)	2.7 (1.3–4.0)	0.1173
Patient survival			
Death from all causes	19 (19.8%)	35 (22.0%)	0.6741
Time-to-death after diagnosis (yrs)	0.9 (0.7–3.0)	1.6 (0.8–2.5)	0.5782
Kidney transplant function			
Returning to hemodialysis	11 (11.5%)	21 (13.2%)	0.7031
Time to resumption of hemodialysis after diagnosis (yrs)	0.9 (0.2–1.4)	1.0 (0.5–1.7)	0.4991

BMI, body mass index; CNI, calcineurin inhibitor; IQR, interquartile range; MMF, mycophenolate mofetil; MPA, mycophenolic acid.

Values are given as number (percentages) or median (IQR).

### Therapeutic Management of Norovirus Infection

At diagnosis, patients with norovirus infection were more frequently treated with belatacept, whereas mammalian target of rapamycin inhibitors were more commonly used in controls (mycophenolate mofetil / mycophenolic acid - belatacept - corticosteroids: 3.1% vs. 20.8%, *P* < 0.0001; mammalian target of rapamycin inhibitors: 15.6% vs. 3.8%, *P* = 0.0012; Table 2).

Immunosuppressive therapy was modified in 120 patients (75.5%) after diagnosis. Among them, 28 (23.3%) discontinued 1 drug class, 40 (33.3%) were switched to an alternative class, and 52 (43.3%) underwent dose reduction. The most frequent change involved withdrawal or reduction of mycophenolate mofetil / mycophenolic acid in 74 (46.5%), occasionally replaced by azathioprine in 21 (13.2%).

Symptomatic antidiarrheal treatment was administered to 54.1% (*n* = 86), whereas only 7.5% (*n* = 12) received oral nutritional supplements. In addition, adjuvant therapies were used in 10.1% (*n* = 16), including nitazoxanide (6.9%), ribavirin (0.6%), polyvalent i.v. Ig (3.1%), and fecal microbiota transplantation (0.6%) (Table 1).

### Follow-Up After Norovirus Infection Diagnosis

At final follow-up, median BMI was 24.3 (IQR: 21.1–27.4) kg/m^2^, representing a 9.1% (IQR: 6.9%–14.3%) increase from diagnosis (*P* < 0.0001). However, among KTRs with severe undernutrition, weight recovery was significantly impaired, with final BMI remaining significantly below baseline (−4%, *P* = 0.0456; Figure 1b). These findings suggest that nutritional status at diagnosis may thus serve as a marker of disease severity, because severe undernutrition was associated with greater initial weight loss and incomplete long-term recovery.

**Figure 2:**
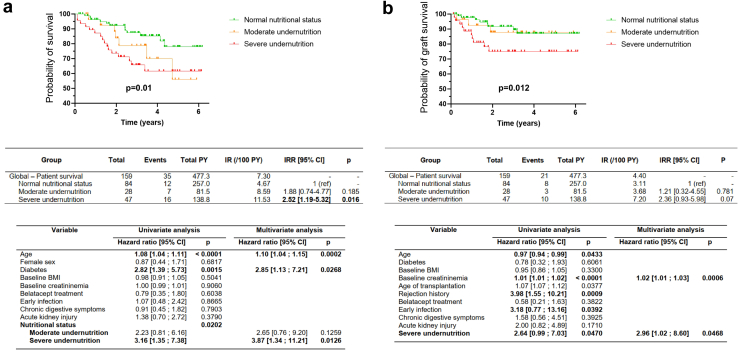
Impact of nutritional status at diagnosis on patient and graft survival in KTRs with norovirus infection. (a) Left panel: Kaplan–Meier survival curves for all-cause mortality among kidney transplant recipients with norovirus infection, stratified by nutritional status at diagnosis. Patient survival was significantly lower in patients with severe undernutrition than in those with moderate or no undernutrition (log-rank trend test, *P* = 0.01). Right panel: IRs and IRRs were calculated using univariable Poisson regression enable subgroup comparisons. Compared with patients without undernutrition, those with severe undernutrition had a significantly increased risk of death (IRR = 2.52 [95% CI: 1.19–5.32], *P* = 0.016). Multivariable Cox analysis identified severe undernutrition (HR = 3.87 [95% CI: 1.34–11.21], *P* = 0.0126), age, and diabetes as independent predictors of mortality. (b) Left panel: Kaplan–Meier survival curves for graft loss (return to dialysis) among kidney transplant recipients with norovirus infection, stratified by nutritional status at diagnosis. Patients with severe undernutrition had a higher risk of graft loss (log-rank trend *P* = 0.012). Right panel: IRs and IRRs were calculated using univariable Poisson regression enable subgroup comparisons. Compared with patients without undernutrition, those with severe undernutrition had a higher risk of graft loss (IRR = 2.32 [95% CI: 0.91–5.91], *P* = 0.07). In multivariable Cox analysis, severe undernutrition (HR = 2.96 [95% CI: 1.02–8.60], *P* = 0.0468) and baseline creatinine predicted graft loss. CI, confidence interval; IR, incidence rate; IRR, incidence rate ratios.

During follow-up, 35 patients (22%) died (Table 1). Kaplan–Meier analysis showed that mortality increased with the severity of undernutrition (log-rank trend *P* = 0.01). Mortality incidence rates increased from 4.7 events per 100 person-years in patients without undernutrition to 8.6 in moderate and 11.5 in severe undernutrition. Compared with patients without undernutrition, the incidence rate ratio for death was 2.52 (95% CI: 1.19–5.32); *P* = 0.016, in severe undernutrition. In multivariable Cox analysis, mortality was independently associated with severe undernutrition (HR = 3.9 [95% CI: 1.34–11.21], *P* = 0.0126), age (HR: 1.10 [95% CI: 1.04–1.15], *P* = 0.0002), and diabetes (HR = 2.85 [95% CI: 1.13–7.21], *P* = 0.0268) (Figure 2a).

Twenty-one patients (13.2%) progressed to dialysis (Table 1). Kaplan–Meier curves showed a higher cumulative incidence of graft loss with increasing severity of undernutrition (log-rank trend *P* = 0.012). Incidence rates for graft loss increased from 3.1 events per 100 person-years in patients without undernutrition to 3.7 in moderate and 7.2 in severe undernutrition. Compared with patients without undernutrition, the incidence rate ratio for graft loss was 2.32 (95% CI: 0.91–5.91); *P* = 0.078, in severe undernutrition. In multivariable analysis, severe undernutrition at diagnosis (HR = 2.96 [95% CI: 1.02–8.60], *P* = 0.047) and baseline serum creatinine (HR = 1.02 [95% CI: 1.01–1.03], *P* = 0.0006) remained independently associated with graft loss (Figure 2b).

Despite immunosuppressive minimization strategies, no significant effect was observed on patient or graft survival, or on rejection incidence (Supplementary Figure S3).

## Discussion

In this retrospective multicentric study of 159 KTRs with norovirus infection compared with 96 nonnorovirus controls, we found a marked nutritional impact, with greater BMI loss and higher rates of severe undernutrition. Severe undernutrition at diagnosis independently predicted mortality and graft loss, underscoring its prognostic significance.

Norovirus infection is a common, late-onset opportunistic complication after transplantation, typically occurring several years posttransplantation. It is associated with concomitant coinfection or viral reactivation in nearly 30% of cases, notably CMV viremia in 15.6%.^8^^,^^16^^,^^17^ In KTRs, diarrhea often causes significant morbidity, including dehydration, drug overexposure, acute kidney injury, and potential allograft rejection.^15^ Norovirus infection can progress to chronic gastroenteritis, with persistent stool viral shedding in 50% of patients.^8^^,^^9^^,^^17^ Unlike previous reports, we found no association between chronic diarrhea at presentation and increased risk of graft loss or mortality.^9^ Instead, nutritional status at diagnosis emerged as a more significant determinant of both patient and kidney allograft outcomes.

Nearly half of the patients presented moderate-to-severe undernutrition. Patients with severe undernutrition at diagnosis had a significantly lower baseline BMI than patients without undernutrition, suggesting that poorer preinfection nutritional status may predispose them to a more severe course of norovirus infection. Compared with matched nonnorovirus controls, norovirus infection was associated with greater chronicity of diarrhea, greater BMI loss, and more frequent severe undernutrition, consistent with prior data showing greater weight loss than other infectious diarrheas (8.6% vs. 3.2%).^8^ Severe undernutrition was associated with the greatest weight loss over time and incomplete recovery by follow-up. These results underscore the specific nutritional impact of norovirus infection, and support the hypothesis that severe undernutrition may not be merely a consequence of protracted norovirus infection, but also a contributing factor to its severity. This dual relationship highlights the need for early nutritional assessment and intervention. Interestingly, it has been previously reported that nutritional status can influence immune response efficiency and the composition of the intestinal microbiota, both of which are critical in resolving gut infections.^24^ Although a trend toward increased mortality with worsening nutritional status was observed in the control cohort, this did not reach statistical significance. In contrast, in the norovirus cohort, the association between severe undernutrition and adverse outcomes was stronger and statistically significant. These findings suggest that though undernutrition represents a general vulnerability factor in KTRs, its prognostic impact may be amplified in the context of norovirus infection.

Currently, no approved antiviral agent or vaccine is available for the treatment of norovirus infection. Management primarily relies on symptomatic interventions and immunosuppressive reduction to facilitate viral clearance. Previous studies have suggested that reducing immunosuppression, particularly by discontinuing mycophenolate, may help resolve symptoms and prevent persistent viral shedding.^17^ In our study, belatacept use was more frequent in the norovirus group than in controls. However, this finding should be interpreted with caution. Previous studies have reported associations between norovirus infection and various immunosuppressive agents, including calcineurin inhibitors, mycophenolate, and lymphocyte-depleting therapies such as antithymocyte globulin.^9^^,^^16^ However, these findings are inconsistent and are likely influenced by confounding factors such as patient characteristics, transplant timing, and center-specific practices. To date, no study has specifically identified belatacept as a risk factor for norovirus infection. A recent study reported a higher incidence of severe infections, including CMV disease, norovirus, and severe COVID-19 infections, in patients receiving belatacept in combination with corticosteroids. In this context, identifying a single causative immunosuppressive agent appears challenging, and the overall intensity of immunosuppression likely plays a key role in viral persistence and disease severity in transplant recipients.^15^ Further studies are needed to better assess the impact of specific regimens and drug combinations in this setting.

Most patients who underwent chronic immunosuppression reduction experienced improvement in gastrointestinal symptoms, but this did not have a significant impact on patient or graft survival. However, these results should be interpreted with caution, because heterogeneity in minimization strategies and the relatively small sample size for subgroup analyses may have limited the statistical power to detect differences in mortality or graft loss. Furthermore, the wide variety of approaches reported in previous studies prevents firm conclusions about the optimal strategy, and immunosuppression reduction carries risks, including the development of donor-specific antibodies and subsequent graft rejection.^16^

This has prompted some groups to explore alternative therapeutic options. Small studies have suggested that nitazoxanide, a broad-spectrum antiprotozoal agent, may reduce the duration and severity of symptoms in norovirus infection.^25^ However, recent studies have cast doubt on these findings.^17^^,^^26^ An early report of a phase 2 prospective, randomized, double-blind study comparing nitazoxanide with placebo for the treatment of norovirus in transplant recipients found that nitazoxanide did not shorten the time to clinical resolution or reduce the duration of viral shedding.^27^ In addition, nitazoxanide does not exhibit selective antiviral activity against norovirus in human small intestinal enteroid models.^28^

The development of an effective vaccine remains a promising strategy for both prevention and treatment of norovirus infection. A recent bivalent vaccine candidate elicited a robust immune response in healthy adults, including a cross-protective humoral and cell-mediated response.^29^ However, several challenges must be addressed, including potential viral escape, the complex relationship between viral diversity and immunity, and the poor correlation between antibody responses and cell-mediated immune responses against norovirus.^30^

In the absence of specific antiviral treatment, symptomatic interventions targeting modifiable risk factors are central to management. Because severe undernutrition is an independent risk factor for both patient and kidney allograft survival, a thorough assessment of nutritional status, including BMI, serum albumin, and prealbumin levels, should be performed at the time of diagnosis and during follow-up. Nutritional support, including a hypercaloric and high-protein diet, may play a crucial role in supporting immunocompromised transplant patients. Oral nutritional supplements and enteral feeding may be necessary in more severe cases. Such interventions should be managed within a multidisciplinary framework, with close collaboration between clinicians and dietitians.

This study has limitations. Its retrospective design has inherent biases, including incomplete data capture and heterogeneity in management. The relatively small sample size, particularly for subgroup analyses, limits the statistical power for certain outcomes, such as the effect of specific immunosuppressive strategies on mortality and graft loss. In addition, although we included a matched control group for the assessment of nutritional status, the 2 cohorts were comparable in terms of demographic characteristics and outcomes, despite some differences in transplant duration and maintenance immunosuppressive regimens.

Despite the limitations and potential biases, our multicenter case series represents the largest study to date on this topic, underscoring the importance of severe undernutrition as an independent risk factor for patient and kidney allograft survival in KTRs with norovirus infection. Close monitoring of nutritional parameters may therefore improve patient outcomes in the absence of antiviral therapies. Further prospective studies are warranted to determine whether active nutritional interventions could improve outcomes in this setting.

## Disclosure

All the authors declared no competing interests.

## Author Contributions

IS did conceptualization, writing, data analyses, and project realization. JZ, CD, HL, MM, RS, and DA participated in conducting the study and writing the paper. MZ did conceptualization, writing, data analyses, and project realization. MD did conceptualization, writing, data analyses, and project realization.

## Supplementary Material

Supplementary File (PDF) **Figure S1.** classification of nutritional stats at diagnosis. **Figure S2.** BMI evolution between baseline and diagnosis in norovirus infected KTRs and KTRs with nonnorovirus related diarrhea. **Figure S3.** (A) Patient survival and (B) kidney allograft survival according to immunosuppression management strategy. **Table S1.** Etiology of diarrhea in norovirus-negative KTR group. **Table S2.** Death and graft loss incidence rates according to nutritional status in norovirus-negative KTR group.

## Footnotes

Supplementary File (PDF)
**Figure S1.** classification of nutritional stats at diagnosis. **Figure S2.** BMI evolution between baseline and diagnosis in norovirus infected KTRs and KTRs with nonnorovirus related diarrhea. **Figure S3.** (A) Patient survival and (B) kidney allograft survival according to immunosuppression management strategy. **Table S1.** Etiology of diarrhea in norovirus-negative KTR group. **Table S2.** Death and graft loss incidence rates according to nutritional status in norovirus-negative KTR group.
