# Supplementary material for: Severe Undernutrition Predicts Adverse Outcomes in Transplant Norovirus Infection
Source: Kidney Int Rep. 2026 Jun 24;11(9):106679. doi: 10.1016/j.ekir.2026.106679 (PMC13396607; doi:10.1016/j.ekir.2026.106679)

Supplementary material:

Table S1: Etiology of diarrhea in norovirus-negative KTR group

	Controls
KTRs	96
**Etiology of diarrhea**	
Non-infectious diarrhea	65.3%
Infectious diarrhea	34.7%
Bacterial (Clostridium difficile and others)	21.2%
Viral (CMV and others)	8.3%
Parasitological	5.2%

Table S2: Death and graft loss incidence rates according to nutritional status in norovirus-negative KTR group

Control Group	Total	Events	IR (/100 PY)	IRR	p-value
**Global - Death**	**96**	**19**	**4.86**		
No undernutrition	64	11	3.88	1 (ref)	
Moderate undernutrition	22	5	6.36	1.64 [0.57–4.72]	0.36
Severe undernutrition	10	3	10.49	2.71 [0.76–9.70]	0.13

Control Group	Total	Events	IR (/100 PY)	IRR	p-value
**Global - Graft loss**	**96**	**11**	**2.81**		
No undernutrition	64	7	2.47	1 (ref)	
Moderate undernutrition	22	3	3.81	1.55 [0.40–5.98]	0.53
Severe undernutrition	10	1	3.50	1.42 [0.17–11.52]	0.74

IR: incidence rates (per 100 patient-years)

IRR: incidence rate ratio

Figure S1: Classification of nutritional stats at diagnosis

	<70 years old patients	>70 years old patients
**Normal nutritional status**	- BMI > 18.5 kg/m^2<br>- And stable BMI or less than 10% decrease<br>- And serum albumin > 35 g/L	- BMI > 22 kg/m^2<br>- And stable BMI or less than 10% decrease<br>- And serum albumin > 30 g/L
**Moderate undernutrition**	- 17 < BMI < 18.5 kg/m^2<br>- Or decrease in BMI > 10% from baseline<br>- Or 30 < serum albumine < 35 g/L	- 20 < BMI < 22 kg/m^2<br>- Or decrease in BMI > 10% from baseline<br>- And serum albumine > 30 g/L
**Severe undernutrition**	- BMI < 17 kg/m^2<br>- Or decrease in BMI > 15% from baseline<br>- Or serum albumine < 30 g/L	- BMI < 20 kg/m^2<br>- Or decrease in BMI > 15% from baseline<br>- Or serum albumine < 30 g/L

Definitions based on 2021 French Authority for Health (HAS) criteria.

Figure S2: BMI evolution between baseline and diagnosis in norovirus infected KTRs and KTRs with non-norovirus related diarrhea

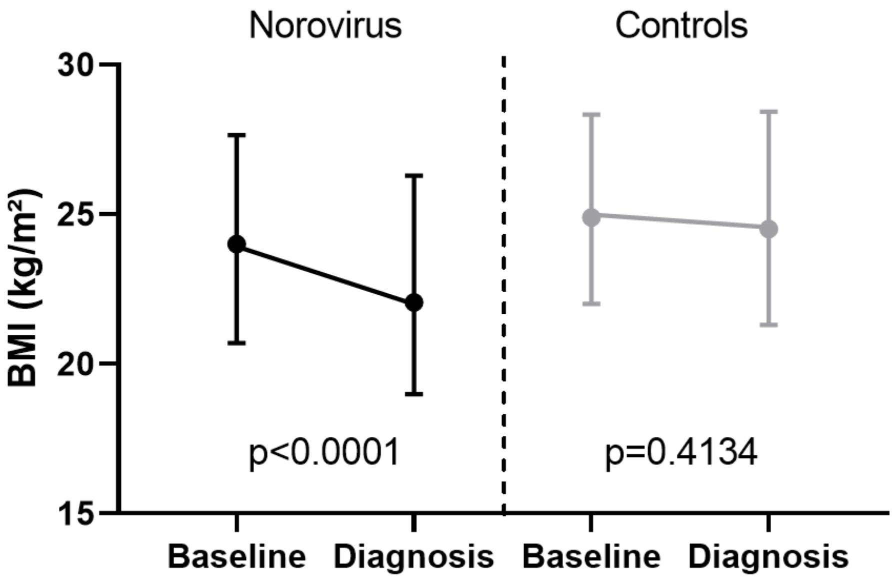

Figure S3: Patient survival (A) and kidney allograft survival (B) according to immunosuppression management strategy

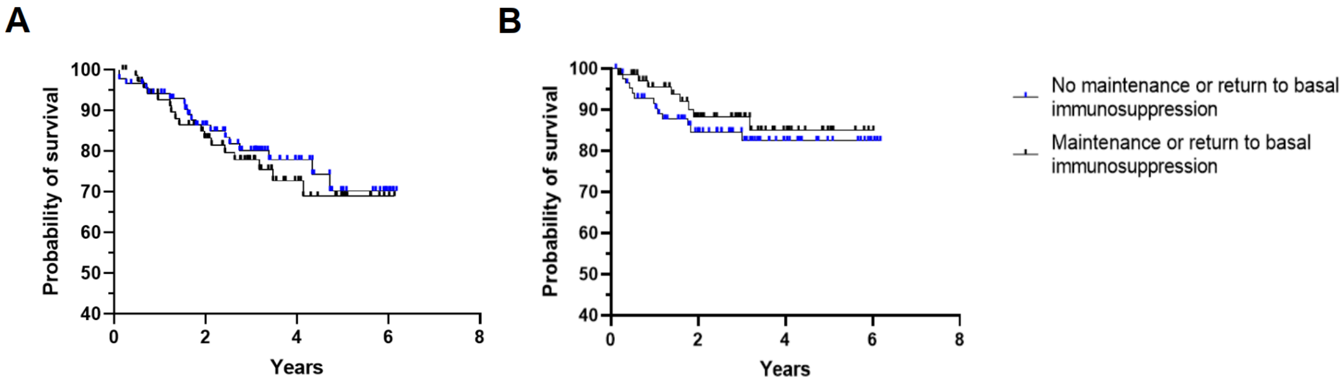

Supplement: Supplementary File (PDF) — Figure S1. classification of nutritional stats at diagnosis. Figure S2. BMI evolution between baseline and diagnosis in norovirus infected KTRs and KTRs with nonnorovirus related diarrhea. Figure S3. (A) Patient survival and (B) kidney allograft survival according to immunosuppression management strategy. Table S1. Etiology of diarrhea in norovirus-negative KTR group. Table S2. Death and graft loss incidence rates according to nutritional status in norovirus-negative KTR group. [file mmc1.pdf]
